# Different Facets of Body Image Disturbance in Binge Eating Disorder: A Review

**DOI:** 10.3390/nu9121294

**Published:** 2017-11-28

**Authors:** Merle Lewer, Anika Bauer, Andrea S. Hartmann, Silja Vocks

**Affiliations:** 1 Department of Clinical Psychology and Psychotherapy, Ruhr-Universität Bochum, Mental Health Research and Treatment Center, Massenbergstr, 9-13, D-44787 Bochum, Germany; 2 Department of Clinical Psychology and Psychotherapy, Universität Osnabrück, Knollstr. 52, D-49069 Osnabrück, Germany; anika.bauer@uni-osnabrueck.de (A.B.); andrea.hartmann@uni-osnabrueck.de (A.S.H.); silja.vocks@uni-osnabrueck.de (S.V.)

**Keywords:** binge eating disorder, body image disturbance, weight and shape concern, checking and avoidance behavior, misperception of body size, body-related cognitive bias

## Abstract

The goal of the present review is to give an overview of the current findings on various facets of body image disturbance in Binge Eating Disorder such as body dissatisfaction, overconcern with weight and shape, body-related checking and avoidance behavior, misperception of body size, and body-related cognitive bias. In addition, treatments for a disturbed body image in BED and evidence of body image disturbance in youth with binge eating are reviewed. The results show that a disturbed body image in BED is present in the form of overconcern with weight and shape. Furthermore, there are hints that body dissatisfaction, as well as body-related checking and avoidance behavior, are also impaired. Research concerning misperception of body size in BED has been neglected so far, but first findings show that individuals with BED rate their own body shape rather accurately. Furthermore, there are first hints that body-related cognitive biases are present in individuals with BED. Moreover, in children and adolescents, there are first hints that body dissatisfaction, as well as shape and weight concerns, seem to be associated with loss of control and binge eating. Treatments aimed directly at the convertibility of a disturbed body image in BED have revealed encouraging outcomes. In conclusion, body image disturbance seems to occur in BED, and first studies show that it can be treated effectively.

## 1. Introduction

In 1994, Binge Eating Disorder (BED) was first introduced as a research category in the Diagnostic and Statistical Manual of Mental Disorders (DSM-IV, [1]). Since BED has proven to be reliably separable from other eating disorders and is associated with clinical levels of eating disorder psychopathology and impairment independent from the existence of comorbid obesity [2], with publication of the DSM-5 [3], it has become a distinct diagnosis of the eating disorder section.

According to the criteria of the DSM-5 [3], BED is defined by episodes of binge eating, during which large amounts of food are consumed within a certain time frame. Simultaneously, the individual experiences a loss of control over the amount and quality of food and the ability to stop the binge eating episode. Further criteria include eating quickly, eating until feeling uncomfortably full, eating alone, disgust or guilt towards oneself and one’s body, feeling ashamed, and eating without being hungry. One binge-eating episode per week for at least three months is needed for diagnosis, and the number of weekly binge eating episodes is used to specify severity. In contrast to other eating disorders, such as Bulimia Nervosa (BN) or the binge eating/purging subtype of Anorexia Nervosa (AN), BED does not comprise any compensatory behaviors such as vomiting, excessive sports, or laxative abuse. Furthermore, as opposed to AN and BN, the criteria for BED do not contain a body image related criterion, such as an overconcern with weight and shape or an undue influence of one’ s own body image on self-worth [3]. However, it remains open to debate whether the inclusion of a respective criterion or specifier might be worthwhile [4].

BED is quite common and is associated with high psychological burden. The lifetime prevalence for BED averages around 2% in various countries [5]. Compared to other eating disorders, the gender ratio is closer to even. Nevertheless, about twice as many women as men suffer from BED [6]. A frequent comorbidity of BED is obesity with associated illnesses such as the metabolic syndrome and type-2-diabetes [7,8,9]. It is assumed that about 65–70% of people fulfilling the criteria for BED also suffer from obesity, defined by the World Health Organization (WHO) as having a body mass index (BMI) of 30 kg/m^2^ or above [7,10,11]. In addition, the proportion of participants with obesity in weight reduction programs, who also suffer from BED, is estimated at 20–30% [12,13]. Concerning mental comorbidities, about 70–79% of patients suffering from BED fulfill the criteria for other mental disorders such as affective disorders and anxiety disorders, and show an elevated risk for suicide, even after controlling for depression [14,15]. Other comorbidities such as substance abuse, posttraumatic stress disorder, body dysmorphic disorder, or personality disorders are also reported, but to a somewhat lesser extent [5].

Although binge eating episodes are already reported in children and adolescents, a full diagnosis of BED in youth is relatively rare, mostly presenting in obese youth seeking weight-loss treatment [16,17]. Binge eating, a combination of eating a perceived large quantity of food accompanied by a sense of a loss of control over eating without fulfilling diagnostic criteria for BED, however, is a common experience [16,18,19,20]. Loss of control (LOC) eating can be even more frequently observed in this age group. Around 9.3% of non-treatment-seeking normal-weight and overweight children aged 6–13 years reported feeling a loss of control over eating, independent of the amount of food eaten [21]. Thus, when examining specific aspects of BED pathology, such as body image disturbance in youth, a wider focus is needed: from LOC eating, to binge eating, to a full diagnosis of BED.

Despite being a fairly “young” disorder, various studies examining etiology of BED have yielded several risk factors. Being overweight or obese during childhood and adolescence, possibly accompanied by teasing and bullying from peers and/or family members, critical life events, neglect, depressiveness or shyness, and physical and sexual abuse are important factors that will lead to an increased vulnerability to the disorder [22,23,24]. Furthermore, factors that are associated with eating disorders, such as emotional eating, restrictive eating in the family, dissatisfaction with one’s own body and figure, dieting, and an internalized slimness ideal are also relevant for the development of BED [23,24,25,26,27].

Besides risk factors, triggering and maintaining factors of BED were identified and integrated in a recent model by Tuschen-Caffier and Hilbert [28]. In this model, the authors describe external and internal stressors such as interpersonal conflicts, exposure to food, impulsiveness, low self-esteem, tension, and an overconcern with one’s weight and figure as triggers for binge eating episodes. Furthermore, overconcern with weight and shape, and body dissatisfaction, are known risk factors for the development of BED (e.g., [29]). In addition, it was shown for adolescent girls that being content with one’s body averted weight gain and binge eating, whereas girls that reported not being satisfied with their bodies tended to gain more weight over time, possibly resulting in being overweight and being teased by others [30]. With regard to other eating disorders such as AN and BN, a disturbed body image is one of the most crucial and prominent risk factors concerning the etiology, maintenance, and relapse of the eating disorder [31,32,33,34]. For example, individuals with AN and BN displayed a greater extent of body dissatisfaction compared to non-eating-disordered females [35,36]. Additionally, several laboratory studies indicated that patients with AN and BN show negative emotions and more negative body-related cognitions when looking at their own bodies than do healthy controls [37,38,39,40]. Moreover, a negative body image not only incorporates body dissatisfaction or a disturbed body image evaluation [41], but also encompasses body-related checking and avoidance behavior [42], a misjudgment of one’s own body size [43], and a body-related attentional bias [37]. Regarding body checking and avoidance behavior, such as frequent weighing, pinching certain body areas, or wearing loose clothing (e.g., [44]), patients with AN or BN were found to show a higher degree of body checking [45,46,47] and avoidance behavior in comparison to healthy individuals [48,49]. The perceptual aspect of body image is described as the mental representation of one’s figure and shape, e.g., the misperception of body size. For example, individuals suffering from AN and BN show a tendency to rather overestimate their own body size [35,50,51,52], whereas individuals without eating disorders seem to slightly underestimate their own body dimensions [43]. The misperception of body size and shape might be related to biased information processing, which is considered to be an important factor in the development and maintenance of body image disturbance in eating pathology [52,53,54,55]. In their cognitive-behavioral model of eating disorders, Williamson and colleagues [56] postulate that different kinds of cognitive biases such as attentional biases, memory biases, and interpretation biases contribute to the distorted body image in individuals with eating disorders. Attentional biases are defined as the selective visual processing of disorder-specific stimuli, e.g., stronger attention allocation towards unattractive as opposed to attractive parts of one’s own body among females with body image disturbance or eating disorders [37,57]. Memory biases are characterized the facilitated encoding and retrieval of disorder-salient information such as weight- and shape-related words or sentences compared to neutral information [58,59]. Interpretation biases refer to the biased processing of ambiguous information, e.g., everyday scenarios, in accordance with disorder-specific cognitive schemata [60].

In view of the prominent role of the different aspects of body image disturbance in AN and BN, it can be hypothesized that body image disturbance might also be a part of the symptomatology of BED [61], although it is not yet included in the diagnostic criteria [3]. In addition, it might prove to be a target for treatment [61,62]. Therefore, the goal of this review is to give an overview of the existing findings concerning the question of whether people suffering from BED also display symptoms of a disturbed body image in terms of body dissatisfaction, an overconcern with weight and shape, body-related checking and avoidance behavior, body size misperception, and body-related cognitive biases. In addition, an outline is drawn of potential treatment strategies targeting body image disturbance in BED and the representation of body image disturbance in youth with BED.

## 2. Body Dissatisfaction

Multiple studies have examined the evaluative aspect of a disturbed body image in BED. One part of this aspect is body dissatisfaction, which is often examined by the Body Dissatisfaction subscale of the Eating Disorder Inventory (EDI, [63]). Various studies have compared participants with BED to participants with BN or, given that the majority of people suffering from BED are at least overweight, to controls with obesity. Some studies reported that individuals with obesity and BED did not score higher on body dissatisfaction than controls with obesity without eating disorders [64,65], thus presumably linking body dissatisfaction to a higher BMI. However, further studies did reveal differences between individuals with BED and overweight or obese and non-clinical individuals with obesity in terms of being less satisfied with their bodies [66,67,68,69,70,71,72], and that participants with BED even scored higher than [65,73] or equal to individuals with BN [74]. In addition, Hilbert and Tuschen-Caffier [75] applied the “think-aloud-technique” during mirror exposure and found that participants with BED and those with BN did not differ in terms of their negative thoughts about their own bodies, but showed significantly more negative body-related cognitions than normal-weight controls, hinting at higher levels of dissatisfaction with their bodies. For further information, see Table 1.

## 3. Overconcern with Weight and Shape

The overconcern with or overvaluation of weight and shape is another aspect of the evaluative facet of body image disturbance, as it not only incorporates dissatisfaction with one’s body but also emphasizes cognitions and emotions regarding one’s own weight and figure and their influence on self-esteem and self-worth [77,78]. Multiple studies have examined the extent to which overvaluation of weight and shape plays a role in the symptomatology of BED, mostly applying the subscales weight concern and shape concern of the Eating Disorder Examination Questionnaire (EDE-Q; [79]), see Table 2. As early as 1993, Spitzer and colleagues [80] compared individuals with obesity with and without BED and found that those with BED were more frequently concerned with body shape and weight than those without BED with a similar BMI, although BED had not yet been introduced as a research category of the DSM [1]. Similar outcomes were reported by subsequent studies [68,81,82,83,84], which also hinted that individuals with BED do not differ from individuals with BN in terms of overvaluation of weight and shape [75,85,86]. Furthermore, various studies have emphasized the importance of overvaluation of shape and weight as a possible specifier for the diagnosis of BED, since individuals suffering from BED, who also report an overconcern with weight and shape, repeatedly display greater eating-disorder pathology, lower self-esteem, and higher depression levels [85,87,88,89,90,91,92]. As Goldschmidt and colleagues [93] were able to show, this aspect seems to be independent of BMI, as they compared individuals with BED who were obese to individuals with BED who were normal-weight regarding weight and shape concern. Moreover, in a treatment study by Grilo, White, Gueorguieva, and colleagues [89], overvaluation of shape and weight significantly predicted non-remission from binge eating at the 12-month follow-up.

## 4. Body-Related Checking and Avoidance Behavior

For body-related checking and avoidance behavior in BED, the current data is fairly limited, as relatively few studies have examined this aspect in BED. Commonly used measurement instruments for the evaluation of body-related checking and avoidance behavior are the Body Checking Questionnaire (BCQ; [46]) and the Body Image Avoidance Questionnaire (BIAQ; [48]), see Table 3. In 2005, Reas, Grilo, Masheb, and Wilson [96] were the first to report that the majority of men and women with BED displayed body checking and avoidance behavior, such as avoiding wearing slim-fitting clothes or pinching areas of one’s own body to check for fatness. However, since the authors did not make use of any control group, it remains unclear whether the scores in terms of checking and avoidance behavior exceed those of a healthy control group. This is in line with another finding by Grilo, Reas, Brody, and colleagues [44], who examined men and women with obesity who were seeking bariatric surgery. The authors demonstrated that body checking and avoidance behavior was a common feature among these patients and was also associated with an overconcern with weight and shape. Unfortunately, as the authors did not perform thorough diagnostics, it remains unclear whether body-related checking and avoidance was attributable to a putative eating disorder or to being extremely overweight with a mean BMI of 51 kg/m^2^. In contrast to the results by Reas et al. [96] and Grilo et al. [44], Lewer and colleagues [68] did not find significant differences between individuals diagnosed with BED and obesity and equal weight controls concerning body checking and avoidance behavior, suggesting that higher scores concerning body-related checking and avoidance behavior are due to obesity rather than being part of BED symptomatology. However, in a recent study, Legenbauer, Martin, and Blaschke et al. [97] found that participants with BED and obesity displayed higher levels of body avoidance than participants with other eating disorders and non-clinical controls, whereas body-related checking was almost absent in this group.

## 5. Body Size Misperception

To date, only three studies have examined this aspect of body image disturbance in BED, which compared individuals who were obese and suffered from BED to equal weight individuals without eating disorders, see Table 4. The results are homogenous in so far as no significant differences were found regarding judgement of body size between the participants with and without BED, although the studies applied different techniques, such as a figure rating scale [71] and a photo distortion technique [67,68]. Interestingly, both studies assessing body size perception with a photo distortion technique revealed that individuals with and without BED estimated their “actual” and “felt” figure rather accurately, demonstrating an almost realistic perception of their body without over- or underestimation. Nevertheless, as Lewer and colleagues showed [68], participants with BED wished for a slimmer “ideal” figure than did the control subjects, hinting at higher levels of dissatisfaction with their current body. In 2011, Nicoli and Liberatore Junior [98] published a study reporting that participants with BED display higher self-image inadequacy than participants without BED, as measured by the figure rating scale. However, as they did not make use of a structured interview for diagnosis, it is difficult to interpret these results as reflecting a body size misperception in BED.

## 6. Body-Related Cognitive Bias

Several studies have underlined the relevance of biased information processing, especially attentional biases, in the field of body image disturbance and eating pathology [52], see Table 5. Although it appears promising to transfer these approaches to BED, little research has been devoted to this field so far.

Referring to the attentional component of body processing, Svaldi, Caffier, and Tuschen-Caffier [99,100] provided evidence to support the occurrence of body-related attentional biases in BED by means of two studies. In the first study, gaze patterns of females with BED and healthy overweight controls were analyzed when looking at photos of their own and a control person’s body. Both groups showed an attentional bias to self-defined unattractive body parts; however, participants with BED looked significantly longer and more often on such body parts than the controls, indicating a specific attentional bias for dissatisfying body areas especially of one’s own body in BED [99]. In the second study by Svaldi and colleagues [100], participants with BED and overweight controls were concurrently presented with own- and other-body pictures in two conditions—either with or without cues indicating the position of one’s own body. In the cue condition, the clinical group showed a significantly higher gaze frequency for own-body pictures and a significantly lower gaze frequency for other-body pictures pointing to a schema-driven pattern of body-related attention allocation in BED [99,100].

First hints of a body-related memory bias in individuals with BED were provided by Svaldi, Bender, and Tuschen-Caffier [76], who conducted a free recall task including positively and negatively valenced body-related or neutral words in females with BED and controls with overweight. Participants of both groups showed a memory bias for negative body-related words; however, the BED group retrieved significantly fewer positive weight/shape cues than the controls, which points to difficulties in processing positive body-related information in BED. Cooper and Wade [60] analyzed the relationship between memory biases and disordered eating behavior in a nonclinical sample of young females, and found a significant correlation between the recall of negative words and the occurrence of binge eating episodes. Furthermore, the authors showed that negative interpretation biases were also associated with binge eating by partially mediating the relationship between difficulties in emotion regulation and overeating [60]. However, as the authors examined a non-clinical sample, results in terms of BED are to be interpreted with caution.

## 7. Treatment of Body Image Disturbance in Binge Eating Disorder

Based on the present literature review, it can be hypothesized that body image disturbance occurs in BED and might consequently be a promising target for treatment. As it has been shown for other eating disorders, treating a disturbed body image has a high impact on recovery and maintenance processes [101,102,103]. To the best of our knowledge, to date, only a small number of studies have explicitly addressed body image disturbance in BED. In one of these studies, which aimed to directly evaluate the effectiveness of distinct interventions targeting body image in BED, it was shown that repeated mirror exposure decreased negative body-related emotions and cognitions, while an increase in appearance self-esteem was observed in a sample of participants with BED and obesity [66]. In a further study, the authors were able to additionally show that body exposure techniques and body image-related cognitive interventions are equally effective in improving body image for individuals with BED and normal weight [103]. Hildebrandt and colleagues [104] examined patients with different eating disorders (AN, BN, BED, eating disorder not otherwise specified) who varied from having a healthy weight to being overweight. The authors applied two treatment designs, namely acceptance-based mirror exposure and a non-directive body image therapy, each comprising five sessions. The results were promising, hinting at a significant superiority of mirror exposure concerning shape and weight concerns and body checking. However, as the overall sample size was rather small and the proportion of participants with BED was only about 6%, conclusions regarding the treatment of BED need to be drawn with caution. In a recent study that explicitly addressed body image disturbance in BED, Lewer and colleagues [62] compared individuals with BED and obesity to a waiting-list control group within a standardized group therapy program consisting of ten sessions. During the program, participants received psychoeducation on the disorder, were guided in cognitive restructuring of negative cognitions towards one’s own body, exposed themselves to their body in the mirror, and received guidance regarding exposure outside of the therapeutic setting (e.g., use personal care products such as body lotion, visit a public pool). The authors found substantial improvements concerning the evaluative and cognition-related aspects of body image disturbance such as significant reductions in body dissatisfaction, weight and shape concerns, drive for thinness, and body-related checking behavior. Furthermore, depressiveness was reduced with a simultaneous improvement in self-esteem. Concerning body size estimation, the authors found that participants rated their “real” and “felt” body image rather accurately at pre- and post-treatment, but wished for a less slim “ideal” figure after treatment. Another recent experimental study assessed the modification of food-related cognitive biases as a further treatment target for BED [105]. The authors showed that food-related attentional bias was successfully reduced by an intervention that used a dot-probe paradigm. Although the reduction of attentional bias lasted only briefly, this design provides first hints for further interventions in the treatment of body image disturbance with BED, for example by using body-related words.

The empirical evidence with regard to the treatment of body image disturbance in BED seems promising. Various studies have demonstrated that different aspects of a disturbed body image can be treated by interventions that explicitly aim at changing one’s own body image. In addition, a number of studies have shown that different treatments for BED exist, such as cognitive-behavioral therapy, interpersonal therapy, and behavioral weight loss therapy, which aim to reduce weight and binge eating symptomatology, but that changes in body image are not always measured directly [89,106,107,108,109,110]. However, as the application of CBT usually incorporates cognitive restructuring, among other interventions, overconcern with weight and shape as part of the evaluative aspect of body image is targeted by some of the interventions at least. For instance, Grilo and colleagues [107] compared CBT to behavioral weight loss treatment (BWL) in a sample of individuals with BED and obesity. They showed that there were no significant differences between the groups concerning the majority of outcome measures, such as overvaluation of weight and shape, general eating disorder pathology, or depression levels. In further examinations, Grilo and colleagues reported that individuals with BED and overvaluation of weight and shape showed greater reductions in eating disorder pathology and depression when receiving CBT versus fluoxetine, and that a rapid response to treatment predicts positive treatment outcome [89,106]. Furthermore, overvaluation of shape and weight significantly predicted non-remission from binge eating in CBT at 12-month follow-up.

## 8. Binge Eating/Loss of Control Eating and Body Image in Youth

To date, very little is known about body image in youth presenting with LOC or binge eating as described above. The identified studies focused solely on the cognitive-affective component of body image. One study found that adolescent individuals presenting overeating with and without LOC had lower body satisfaction assessed with a modified version of the Body Shape Satisfaction Scale [111] compared to individuals without overeating [112]. Several studies examined thoughts and attitudes towards one’s body, mostly reported as shape and weight concerns in young individuals with LOC or binge eating. A study by Tanofsky-Kraff and colleagues [21] reported greater shape and weight concerns in 6–13-year-olds in the normal weight and overweight categories with LOC eating compared to those without LOC eating. This finding was confirmed in a study by Goossens et al. [19], who found elevated shape and weight concerns in overweight 10–16-year-olds with LOC eating compared to those without LOC eating. A more recent study found corresponding differences in youth with and without LOC eating post-puberty but not pre-puberty [113]. Geller and colleagues [114] showed that overvaluation of shape and weight is associated with eating disorder symptoms in general in girls aged 13–18-year-old girls. Both Goldschmidt and colleagues [93] and Hilbert and Czaja [115] found that overvaluation was associated with greater eating disorder pathology (e.g., frequency of LOC eating episodes). Hilbert and Czaja [115] further identified the importance of shape and weight, assessed with the corresponding item from the child version of the Eating Disorder Examination and serving as a proxy for overvaluation to have high discriminatory power (i.e., leading to greater specificity in detecting clinically significant cases of LOC eating).

Distinctive characteristics within the cognitive and affective component are not only present in youth with LOC or binge eating—they also seem to be predictive for the incidence or course. In two studies, Goldschmidt and colleagues showed that besides other variables, body dissatisfaction or the increase thereof predicted binge eating during adolescence and young adulthood [112,116]. With regard to the cognitive component, Neumark-Sztainer et al. [117] found that in treatment-seeking adolescents who were overweight, weight concerns predicted the incidence of binge eating over a 5-year period. Another study confirmed the predictive value of both components, demonstrating that a combination of appearance overvaluation, body dissatisfaction, depressive symptoms, BMI, and dieting predicted the incidence of binge eating over an approximately two-year follow-up in adolescent girls [29].

Thus, in sum, body dissatisfaction and shape and weight concerns (in particular the overvaluation thereof) seem to be associated with LOC eating and binge eating in youth, although no distinct BED diagnoses were allocated. There is evidence that this might, besides other variables, even be predictive for incidence or course of LOC or binge eating. An examination of the perceptual, as well as the behavioral, component of body image is lacking and is needed in order to fully understand the body image of youth with LOC or binge eating.

## 9. Discussion

The results of the present narrative review support the hypothesis that body image disturbance occurs in BED. Particularly with regard to an overconcern with weight and shape, there is strong support for this aspect to be present in BED, similar to other eating disorders [61,68,81,85]. Furthermore, overvaluation of shape and weight is associated with higher levels of eating disorder pathology, depression, and lower self-esteem [90,92]. Although it occurs in the majority of individuals with BED, there is a subgroup of the BED population that does not report overvaluation of shape and weight as part of their pathology [77,85,92,94,118]; consequently, the proposed implementation of overvaluation of shape and weight as a diagnostic specifier [4] seems reasonable. Furthermore, there is evidence that applying overvaluation of shape and weight as a diagnostic specifier provides stronger information about eating disorder psychopathology than the proposed severity rating in the DSM-5 [88]. The outcomes concerning body dissatisfaction also point to higher levels of body dissatisfaction in individuals with BED compared to controls without eating disorders. However, evidence is not explicitly clear, since some results indicate a link between a higher BMI and body dissatisfaction independent of BED symptomatology. This might result from the way body dissatisfaction is examined, since the body dissatisfaction subscale from the EDI [63], which was applied in most of the studies, was originally designed for underweight to normal-weight populations of individuals with AN and BN and might fail to detect differences in samples of overweight or obese participants. Furthermore, there are initial hints that BED is also associated with enhanced body-related checking and avoidance behavior. However, it needs to be taken into consideration that this aspect of body image disturbance has been somewhat neglected in BED, and the studies targeting body-related checking and avoidance behavior reviewed in this article reached inconsistent results. In terms of body size misperception, the empirical evidence with regard to BED is similarly low to that for the behavioral facet. Nevertheless, most studies conclude that individuals with BED rate their own body size rather realistically, without over- or underestimation [67,68], which is not in line with research concerning AN and BN that has revealed that individuals with AN and BN rather overestimate their body size [35,50,52]. In addition, research on cognitive biases in BED is still in its infancy, but there are first hints of a biased processing of body cues on body image in BED [76,100], with a bias towards the self-defined, most unattractive body parts when looking at one’s own body.

As Williamson et al. [56] proposed in their cognitive-behavioral model of eating disorders, overconcern with weight and shape might function as a psychological risk factor for a self-image relating unduly to body size and shape. As suggested by the results of the present review, this might also be applicable for individuals with BED, since an overconcern with weight and shape seems to be present in the disorder, which might further lead to cognitive biases such as overestimation of body size and memory, as well as attentional biases and behavior such as body-related checking and avoidance behavior. As the empirical evidence referring to these aspects is still relatively scarce for individuals with BED, it is not yet clear whether individuals with BED are also influenced by cognitive biases, body size misperception, or checking and avoidance behavior. Nevertheless, Williamson et al. [56] propose that a cognitive bias leads to increased negative emotions and dietary restraint, which is both common in individuals with BED [62,67,68,87] and in turn might lead to binge eating behavior. Furthermore, there are signs that overvaluation of weight/shape triggered by body-related stimuli, such as watching other persons in a fashion store, might further increase the desire to binge in individuals with BED [119]. As a consequence of binge eating and cognitive biases, overconcern with shape and weight is confirmed, along with other aspects such as internalization of a thin ideal, which has been proven a predictor for the onset of BED [27]. Williamson and colleagues [56] assume that these aspects might lead to a reduction of negative emotions in the short term, but might stabilize a self-schema that relates to body size and shape or eating in the long term. For a more comprehensive understanding of body image disturbance, the empirical evidence to date suggests that the cognitive-behavioral model by Williamson et al. [56] might also apply to BED. However, aspects of body image disturbance in BED other than overconcern with weight and shape have not yet been sufficiently analyzed. Studies are needed that examine the different aspects of body image in BED with higher sample sizes in order to obtain a sufficient statistical power, as well as in diverse samples. Most studies, for instance, have assessed individuals with BED and comorbid obesity, although there is also a subgroup of individuals with BED who are of normal weight. Although the severity of psychopathology in BED does generally not seem to be associated with BMI, Dingemans and Van Furth [120] reported that individuals with BED and comorbid obesity display a higher binge-eating frequency and greater levels of weight concern than individuals with BED and normal weight. However, despite the fact that obesity is the most common comorbidity of BED [5,7], the causality of BED and obesity is not yet clear. Are binge-eating episodes the reason for becoming overweight or obese and then being dissatisfied with one’s body, or is obesity itself associated with elevated emotional distress and might eventually lead to binge-eating as a coping mechanism [121,122]? There are signs that being overweight during childhood is a risk factor for the development of the disorder [24] and that body dissatisfaction and shape and weight concerns already seem to be associated with LOC eating and binge eating in adolescence [112,117].

Concerning the treatment of body image disturbance in BED, various studies have compared CBT, IPT, and BWL as interventions targeting binge-eating symptomatology (e.g., [106,108,110,123,124]). With regard to body image disturbance, overconcern with weight and shape seems to be responsive to CBT, which mostly incorporates cognitive restructuring techniques addressing potentially dysfunctional cognitions among others regarding one’s own body, figure, and self-worth. Only a small number of studies to date have explicitly aimed at changing a disturbed body image in BED [62,66,103]. The results of these studies are promising, as they seem to impact at least the evaluative aspect of body image, such as overconcern with shape and weight and body dissatisfaction, but also provide promising evidence concerning the reduction of body-related checking behavior, body-related eating-disorder pathology, and depression levels, as well as the improvement of self-esteem. Regarding the misperception of the size of one’s own body, it was shown that participants with BED and obesity rate their actual and felt figure rather accurately [67,68], but there is evidence that participants wished for a less slim ideal figure after treatment [62]. This is of note, since most treatment studies report a modest weight reduction in participants with BED at best [107,109], with unclear effects concerning weight in the long-term [125,126]. Although greater thin ideal internalization predicts onset of BED [27], to our knowledge, no previous study has compared thin ideals in individuals with various eating disorders, including BED and individuals without eating disorder. Research assessing the thin ideals of individuals with BED compared to those in individuals with AN, BN, and healthy controls might help to clarify the relationship with ideal body shape, binge eating, and potential weight loss. In sum, treatments incorporating cognitive-behavioral techniques or weight reduction programs both seem to be somewhat effective in BED, and treatments that directly aim at changing a disturbed body image in BED show promise.

The goal of the present narrative review was to provide an overview of the current empirical evidence concerning body image disturbance in BED. Various aspects of body image disturbance such as overconcern with weight and shape, body dissatisfaction, probably body-related checking, and avoidance behavior occur in BED. Furthermore, there are signs of the existence of body-related cognitive biases in BED. However, future studies directly comparing individuals with BED with and without obesity with individuals with AN and BN within one study would be helpful to validate whether aspects of body image disturbance in BED occur to a similar degree as in other eating disorders. Furthermore, many of the reviewed studies examined individuals with BED and comorbid obesity, so data concerning overweight and normal-weight patients with BED is missing. A joint limitation, especially of the studies regarding body-related checking and avoidance behavior, misperception of body size, and body-related cognitive bias, is the small sample size; as a consequence of this, possible effects might not have been detected due to a small power. Moreover, future studies are needed that include males and children and adolescents with BED/LOC, since research with male participants has been somewhat neglected and empirical evidence on youth is still at an early stage. With regard to treatment of BED, interventions such as, especially, CBT and IPT seem to be successful in the short term, but with unclear results concerning long-term effects. In addition, treatments focusing directly on changing a disturbed body image shows promise but needs to be replicated with larger samples, untreated control groups, and long-term follow-up intervals. While the role of body image disturbance in terms of etiology, maintenance, and relapse processes has been documented in AN and BN [31,33,127], the empirical evidence concerning this aspect in BED is still rather scarce. Nevertheless, it has been shown that body dissatisfaction is a risk factor for the onset of binge eating and is already present in children and adolescents [21,29,30]. Moreover, overvaluation of shape and weight has been found to be an important parameter concerning remission from binge eating after treatment [89]. Concerning the other facets of body image disturbance in BED, research on their predictive power regarding the development and maintenance of eating disorder pathology and response to treatment is still missing. Furthermore, it remains unclear whether the various components of body image disturbance in BED are connected. Nevertheless, there are signs that overconcern with weight and shape is associated with higher levels in body-related checking and avoidance behavior in BED [67], but studies correlating aspects of body image disturbance in BED with eating disorder pathology such as binge eating frequency are needed to quantify the importance of body image disturbance in BED. Accordingly, treatments designed explicitly for BED, and which do not merely involve transfer from interventions from the treatment of other eating disorders, can be developed.

In conclusion, the findings of this narrative review suggest that body image disturbance occurs in BED, especially in terms of overconcern with weight and shape and body dissatisfaction. Results concerning the other aspects of body image disturbance such as body-related checking and avoidance behavior, misperception of body size, and body-related cognitive bias are still relatively scarce, but also point towards a disturbed body image in BED. Furthermore, there are signs that body image disturbance in BED is positively responsive to treatment.

## Figures and Tables

**Table 1:** Studies of body dissatisfaction.

Authors	Participants	Measures	Results
Kuehnel & Wadden (1994) [65]	*N* = 11 obese participants with BED *N* = 30 obese participants without BED *N* = 29 “problem eaters” (who reported binge eating but did not meet other criteria for BED) (BMI: *M =* 37 kg/m^2^)	Eating Disorder Inventory-2	No differences were found between the three groups on the body dissatisfaction subscale.
De Zwaan et al. (1994) [64]	*N* = 43 obese participants with BED *N* = 22 obese participants without BED (BMI: *M* = 35.9 kg/m^2^)	Eating Disorder Inventory	No differences were found between obese participants with BED and the group without BED on the body dissatisfaction subscale.
Raymond et al. (1995) [73]	*N* = 43 obese participants with BED (BMI: *M* = 36 kg/m^2^) *N* = 35 participants with BN (BMI: *M* = 22 kg/m^2^)	Eating Disorder Inventory	Participants with BED scored higher on the body dissatisfaction subscale than participants with BN.
Sorbara & Geliebter (2002) [71]	*N* = 38 obese participants with BED *N* = 20 obese participants without BED (BMI: *M* = 37 kg/m^2^)	Eating Disorder Inventory	Participants with BED scored higher on the body dissatisfaction subscale than participants without BED.
Barry et. al. (2003) [74]	*N* = 79 obese participants with BED (BMI: *M* = 39 kg/m^2^) *N* = 37 non-obese participants with BED (BMI: *M* = 26 kg/m^2^) *N* = 46 participants with BN (BMI: *M* = 23 kg/m^2^)	Eating Disorder Inventory	No differences were found between the three groups on the body dissatisfaction subscale.
Svaldi, Bender, Tuschen-Caffier (2010) [76]	*N* = 18 obese participants with BED (BMI: *M* = 32.8 kg/m^2^) *N* = 18 obese participants without BED (BMI: *M* = 30.7 kg/m^2^)	Recall-Task (positive body-related words, positive control words, negative body-related words, negative control words)	Participants with BED retrieved positive body-related words significantly less often than the control group.
Legenbauer et al. (2011) [67]	*N* = 15 obese participants with BED *N* = 15 obese participants without BED (BMI: *M* = 43 kg/m^2^)	Eating Disorder Inventory-2	Obese participants with BED scored significantly higher than obese participants without BED on the body dissatisfaction subscale.
Vinai, Da Ros, Speciale et al. (2014) [72]	*N* = 57 obese participants with BED *N* = 61 obese participants without BED (BMI: *M* = 45 kg/m^2^)	Eating Disorder Inventory	Participants with BED scored significantly higher on drive for thinness, bulimia, body dissatisfaction, and interoceptive awareness.
Lewer, Nasrawi, Schroeder et al. (2015) [68]	*N* = 31 obese participants with BED (BMI: *M* = 35.8 kg/m^2^) *N* = 28 obese participants without BED (BMI: *M* = 37.2 kg/m^2^)	Eating-Disorder-Inventory-2 Eating Disorder Questionnaire	Participants with BED displayed significantly higher levels of drive for thinness, body dissatisfaction, and weight and shape concerns.

Note: AN = Anorexia Nervosa; BMI = Body Mass Index; BED = Binge Eating Disorder; BN = Bulimia Nervosa; EDNOS = Eating disorder not otherwise specified.

**Table 2:** Studies of overconcern with weight and shape.

Authors	Participants	Measures	Results
Eldredge & Agras (1996) [82]	*N* = 35 obese participants with BED *N* = 68 obese participants without BED *N* = 53 participants with EDNOS (BMI: *M* = 30 kg/m^2^)	Eating Disorder Examination Questionnaire	Participants with BED scored higher than obese participants without BED on the shape and weight concern subscale.
Nauta et al. (2000) [84]	*N* = 37 obese participants with BED *N* = 37 obese participants without BED (BMI: Range = 27 to 45 kg/m^2^)	Eating Disorder Examination Questionnaire	Participants with BED scored higher than obese participants without BED on the shape and weight concern subscale.
Allison et al. (2005) [81]	*N* =177 obese participants with BED *N* = 68 obese participants without BED	Eating Disorder Examination	Participants with BED scored higher than overweight controls on the shape and weight concerns subscale.
Hilbert et al. (2005) [75]	*N* = 30 participants with BED (BMI: *M* = 27 kg/m^2^) *N* = 30 participants with BN (BMI: *M* = 23 kg/m^2^) *N* = 30 normal-weight controls (BMI: *M* = 25 kg/m^2^)	Think Aloud Technique during body exposure Eating Disorder Examination	There were no differences between participants with BED and those with BN on the subscales shape and weight concern. Participants with BN and BED more frequently had negative cognitions concerning their own body than normal-weight controls.
Mond et al. (2006) [92]	*N* = 51 participants with BED with extreme weight and shape concerns (BMI: *M* = 32 kg/m^2^) *N* = 59 participants with BED without extreme weight and shape concerns (BMI: *M* = 25 kg/m^2^) *N* = 128 participants with an eating disorder (BMI: *M* = 23 kg/m^2^) *N* = 457 obese participants without BED (BMI: *M* = 35 kg/m^2^)	Eating Disorder Examination Questionnaire	Participants with BED who had extreme weight and shape concerns had significantly higher levels of eating disorder, psychopathology, and functional impairment than those without shape and weight concerns.
Grilo et al. (2008) [87]	*N* = 92 participants with BED with clinical overvaluation (BMI: *M* = 37 kg/m^2^) *N* = 73 participants with BED with subclinical overvaluation (BMI: *M* = 36 kg/m^2^) *N* = 45 obese participants without BED (BMI: *M* = 37 kg/m^2^)	Eating Disorder Examination	Participants with overvaluation of shape and weight had significantly higher scores in eating disorder pathology and depression than those with subclinical levels of overvaluation. Both BED groups had higher eating disorder pathology and depression scores than the group without BED.
Grilo et al. (2010) [77]	*N* = 101 participants with BED who overvalue weight and shape (BMI: *M* = 36 kg/m^2^) *N* = 47 participants with BED who do not overvalue weight and shape (BMI: *M* = 35 kg/m^2^) *N* = 53 participants with BN (BMI: *M* = 33 kg/m^2^) *N* = 123 obese participants without BED (BMI: *M* = 33 kg/m^2^)	Eating Disorder Examination Questionnaire	Participants with BED had higher eating disorder pathology than the obese group without BED. Participants with BED who overvalue shape and weight showed more eating disorder pathology and depression levels than BED participants who do not overvalue shape and weight. The BED group with overvaluation and the BN group did not differ significantly from each other.
Goldschmidt, Le Grange, Powers et al. (2011) [94]	*N* = 86 normal-weight participants with BED (BMI: *M* = 22.0 kg/m^2^) *N* = 195 obese participants with BED (BMI: *M* = 42.2 kg/m^2^)	Eating Disorder Questionnaire	The groups did not differ concerning overvaluation of shape and weight.
Grilo, White & Masheb (2012) [90]	*N* = 97 participants with BED with overvaluation *N* = 45 participants with BED without overvaluation (BMI: *M* = 38.5 kg/m^2^)	Eating Disorder Examination Interview	Participants with BED and clinical overvaluation of shape and weight had greater levels of eating-disorder pathology, lower self-esteem, higher depression levels, and more comorbid anxiety disorders.
Grilo, White, Gueorguieva et al. (2013) [89]	*N* = 52 participants with BED with overvaluation *N* = 38 participants with BED without overvaluation (BMI: *M* = 38.7 kg/m^2^)	Eating Disorder Examination Interview	Participants with BED and overvaluation of shape and weight had greater levels of eating-disorder pathology and lower self-esteem. Overvaluation predicted non-remission from binge-eating.
Naumann, Trentowska & Svaldi (2013) [83]	*N* = 43 participants with BED and obesity (BMI: *M* = 35 kg/m^2^) *N* = 44 participants with obesity but without BED (BMI: *M* = 34 kg/m^2^)	Eating Disorder Examination Questionnaire	Participants with BED report significantly higher weight and shape concerns than controls.
Grilo, Ivezaj & White (2015) [88]	*N* = 834 participants with BED (BMI: *M* = 38.0 kg/m^2^)	Eating Disorder Examination Interview	Participants with BED and overvaluation of shape and weight had greater levels of eating-disorder pathology and depression levels.
Harrison, Mond, Rieger et al. (2015) [91]	*N* = 37 participants with BED with overvaluation (BMI: *M* = 29.8 kg/m^2^) *N* = 78 participants with BED without overvaluation (BMI: *M* = 26.2 kg/m^2^) *N* = 194 obese participants without BED (BMI: *M* = 35.2.8 kg/m^2^) *N =* 78 normal-weight participants without BED (BMI: *M* = 22.2 kg/m^2^)		Participants with BED and overvaluation of shape and weight had greater levels of eating-disorder pathology, general psychological distress, and poorer psychosocial functioning than participants with BED without overvaluation. The obese participants without BED did not differ from participants with BED without overvaluation of shape and weight on any outcome measure.
Becker & Grilo (2015) [95]	*N =* 347 participants with BED *(N* = 129 participants with a comorbid mood disorder (BMI: *M* = 38.6 kg/m^2^) *N* = 34 participants with a comorbid substance use disorder (BMI: *M* = 37.8 kg/m^2^) *N* = 60 participants with a comorbid mood and substance use disorder (BMI: *M* = 37.7 kg/m^2^) *N* = 124 participants without a comorbid mood and substance use disorder (BMI: *M* = 36.8 kg/m^2^)	Eating Disorder Examination	The participants with comorbid disorder displayed higher weight and shape concerns.
Lewer, Nasrawi, Schroeder et al. (2015) [68]	*N* = 31 obese participants with BED (BMI: *M* = 35.8 kg/m^2^) *N* = 28 obese participants without BED (BMI: *M* = 37.2 kg/m^2^)	Eating-Disorder-Inventory-2 Eating Disorder Questionnaire	Participants with BED displayed significantly higher levels of drive for thinness, body dissatisfaction, and weight and shape concerns.

Note: AN = Anorexia Nervosa; BMI = Body Mass Index; BED = Binge Eating Disorder; BN = Bulimia Nervosa; EDNOS = Eating disorder not otherwise specified.

**Table 3:** Studies of body-related checking and avoidance behavior.

Authors	Participants	Measures	Results
Reas et al. (2005) [96]	*N* = 377 participants with BED (BMI > 25 kg/m^2^)	Body Shape Questionnaire	The majority of the participants with BED reported body checking and avoidance behavior.
Lewer, Nasrawi, Schroeder et al. (2015) [68]	*N* = 31 obese participants with BED (BMI: *M* = 35.8 kg/m^2^) *N* = 28 obese participants without BED (BMI: *M* = 37.2 kg/m^2^)	Body Image Avoidance Questionnaire Body Checking Questionnaire	The groups did not differ concerning body image avoidance or body checking behavior.
Legenbauer, Martin, Blaschke et al. (2017) [97]	*N =* 30 participants with BED (BMI: *M* = 35.1 kg/m^2^) *N* = 71 participants with AN (BMI: *M* = 16.7 kg/m^2^) *N* = 178 participants with BN (BMI: *M* = 22.0 kg/m^2^) *N* = 31 participants with EDNOS (BMI: *M* = 27.0 kg/m^2^) *N* = 112 non clinical controls (BMI: *M* = 21.9 kg/m^2^)	Eating Disorder Examination Questionnaire Eating Disorder Inventory-2 Body Image Avoidance Questionnaire Body Checking Questionnaire	Participants with BED reported the highest levels of body image avoidance compared to all other groups.

Note: AN = Anorexia Nervosa; BMI = Body Mass Index; BED = Binge Eating Disorder; BN = Bulimia Nervosa; EDNOS = Eating disorder not otherwise specified.

**Table 4:** Studies of misperception of body size.

Authors	Participants	Measures	Results
Sorbara & Geliebter (2002) [71]	*N* = 38 obese participants with BED *N* = 20 obese participants without BED (BMI: *M* = 37 kg/m^2^)	Figure Rating Scale	Females with and without BED did not differ in the estimation of their own body size.
Legenbauer et al. (2011) [67]	*N* = 15 obese participants with BED *N* = 15 obese participants without BED (BMI: *M* = 43 kg/m^2^)	Photo Distortion Technique	There were no significant differences between obese participants with BED and obese participants without BED.
Lewer, Nasrawi, Schroeder et al. (2015) [68]	*N* = 31 obese participants with BED (BMI: *M* = 35.8 kg/m^2^) *N* = 28 obese participants without BED (BMI: *M* = 37.2 kg/m^2^)	Photo Distortion Technique	Participants with BED whose “ideal” figure was significantly slimmer than that of participants without BED. The groups did not differ concerning their perceived “actual” and “felt” figure.
Nicoli & Liberatore Junior (2011) [98]	*N* = 28 participants with BED *N* = 189 participants without BED (BMI: not specified or both groups)	Binge Eating Scale Figure Rating Scale	Participants with BED showed higher self-image inadequacy than participants without BED.

Note: AN = Anorexia Nervosa; BMI = Body Mass Index; BED = Binge Eating Disorder; BN = Bulimia Nervosa; EDNOS = Eating disorder not otherwise specified.

**Table 5:** Studies of body-related cognitive bias.

Authors	Participants	Measures	Results
Cooper & Wade (2015) [60]	*N* = 181 obese and non-obese participants (non-clinical) (BMI: *M* = 22.99 kg/m^2^)	Recall-Task (positive trait adjectives, negative trait adjectives, neutral words) Ambiguous Scenarios Test for Depression	The recall of negative words and the occurrence of binge eating episodes were significantly correlated. Negative interpretation biases were associated with binge eating by partially mediating the relationship between difficulties in emotional regulation and overeating.
Svaldi, Bender & Tuschen-Caffier (2010) [76]	*N* = 18 obese participants with BED (BMI: *M* = 32.8 kg/m^2^) *N* = 18 obese participants without BED (BMI: *M* = 30.7 kg/m^2^)	Recall-Task (positive body-related words, positive control words, negative body-related words, negative control words)	Participants with BED retrieved positive body-related words significantly less often than the control group.
Svaldi, Caffier & Tuschen-Caffier (2011) [99]	*N* = 26 participants with BED (BMI: *M* = 38.7 kg/m^2^) *N* = 18 obese participants without BED (BMI: *M* = 30 kg/m^2^)	Eye-tracking experiment (photos of one’s own and a BMI-matched control person’s body)	Participants with BED looked significantly longer and more often on self-defined unattractive body parts than the control group.
Svaldi, Caffier & Tuschen-Caffier (2012) [100]	*N* = 23 participants with BED (BMI: *M* = 37.7 kg/m^2^) *N* = 23 obese participants without BED (BMI: *M* = 29.8 kg/m^2^)	Eye-tracking experiment (photos of one’s own and a weight-matched control person’s body which were presented with or without cues indicating the position of one’s own body)	In the cue-condition participants with BED showed a significantly higher gaze frequency for self-body pictures and a significantly lower gaze frequency for other-body pictures. In the no-cue-condition no significant group differences were found concerning the attentional patterns.

Note: AN = Anorexia Nervosa; BMI = Body Mass Index; BED = Binge Eating Disorder; BN = Bulimia Nervosa; EDNOS = Eating disorder not otherwise specified.

## Abbreviations

BED	Binge Eating Disorder
AN	Anorexia Nervosa
BN	Bulimia Nervosa
DSM	Diagnostic and Statistical Manual of Mental Disorders
EDE	Eating Disorder Examination Interview
EDI	Eating Disorder Inventory
LOC	Loss of Control
CBT	Cognitive Behavioral Therapy
IPT	Interpersonal Therapy
BWL	Behavioral Weight Loss Therapy
